# PBX1 as Pioneer Factor: A Case Still Open

**DOI:** 10.3389/fcell.2017.00009

**Published:** 2017-02-14

**Authors:** Britta M. Grebbin, Dorothea Schulte

**Affiliations:** Institute of Neurology (Edinger Institute), University Hospital Frankfurt, J. W. Goethe UniversityFrankfurt, Germany

**Keywords:** TALE homeodomain protein, PBX, pioneer factor, adult stem cell, cell fate specification, chromatin remodeling

## Abstract

Pioneer factors are proteins that can recognize their target sites in barely accessible chromatin and initiate a cascade of events that allows for later transcriptional activation of the respective genes. Pioneer factors are therefore particularly well-suited to initiate cell fate changes. To date, only a small number of pioneer factors have been identified and studied in depth, such as FOXD3/FOXA1, OCT4, or SOX2. Interestingly, several recent studies reported that the PBC transcription factor PBX1 can access transcriptionally inactive genomic loci. Here, we summarize the evidence linking PBX1 with transcriptional pioneer functions, suggest potential mechanisms involved and discuss open questions to be resolved.

## Transcriptional pioneer factors

Transcriptional activation or repression of tissue specific genes is typically controlled by a set of sequence specific transcription factors (TFs), together with additional co-regulatory proteins. In addition, there is accumulating evidence that efficient *de novo* activation of previously silent genes often also depends on DNA–protein interactions and chromatin modifications that occur long before mRNA transcripts of these genes can be detected. These processes are carried out by a special type of DNA binding proteins, termed pioneer TFs (Smale, 2010; Iwafuchi-Doi and Zaret, 2014). Two of the first identified and best studied pioneer factors are the FOX family members FOXD3 and FOXA1. FOXD3 is bound to an enhancer of the liver specific *albumin* (*Alb1*) gene already in embryonic stem cells (ESCs), although the gene is not transcriptionally activated until hepatocyte differentiation (Gualdi et al., 1996; Bossard and Zaret, 1998; Xu et al., 2007). During endodermal differentiation, FOXD3 gets downregulated and FOXA1 becomes upregulated and subsequently binds this *Alb1* enhancer together with GATA-4 (Gualdi et al., 1996; Bossard and Zaret, 1998). FOXD3 thus serves as a placeholder for FOXA1 and both proteins provide an early molecular anchor for other TFs at the *Alb1* enhancer, facilitating later *Alb1* gene activation upon differentiation into hepatocytes (Smale, 2010).

Since the 1990s, a number of additional pioneer TFs were discovered. The emerging definition of a pioneer factor comprises the ability to (1) engage its target site(s) in closed chromatin prior to gene activation, (2) increase chromatin accessibility for other proteins at this site, and (3) establish competence for cell fate changes and thus play a key role in cellular (re-) programming (Iwafuchi-Doi and Zaret, 2014). For about two decades, the pressing question of how pioneer factors can access genomic sites in silent chromatin remained a largely unresolved issue, yet recent structural investigations from the Zaret laboratory provide novel advances in the understanding of possible binding modes of pioneer factors to nucleosomal DNA (Soufi et al., 2015). Previously, it was postulated that pioneer factors may access their binding sites in compacted chromatin because of a local destabilization of the nucleosome-DNA contact. Suggested mechanisms of destabilization were the presence of poly(dA-dT) sequences or of histone variants such as H3.3 or H2A.Z (Sekinger et al., 2005; Zhang et al., 2005; Jin et al., 2009). However, genome-wide approaches found that many regulatory regions do not coincide with DNA sequences that assemble into unstable nucleosomes (Zaret and Carroll, 2011). Instead, there is accumulating evidence that pioneer TFs can recognize their target DNA binding sites even in compacted chromatin. The prototype pioneer factor FOXA1, for instance, associates with DNA through a “winged helix”-type DNA binding domain, which structurally resembles the linker histones H1 and H5 (Clark et al., 1993; Ramakrishnan et al., 1993). The FOXA1 C-terminus, on the other hand, can bind to core histones independently of the protein's DNA-binding domain (Cirillo et al., 2002; Sekiya et al., 2009). These structural characteristics enable FOXA1 to penetrate nucleosomal chromatin and, by competition, to displace linker histones. FOXA1 thereby paves the way for other TFs to bind. Consequently, pioneer factors, like linker histones, are retained on mitotic chromosomes and thus might assume a “bookmarking” function during mitosis (Yan et al., 2006; Taube et al., 2010; Zaret et al., 2010; Iwafuchi-Doi et al., 2016). However, not all known pioneer factors possess such linker histone-like properties or winged helix motifs. For example, the reprogramming factors OCT4, SOX2, and KLF4 share the ability to target partial recognition motives on nucleosomes where only one face of the DNA is accessible, yet possess very different DNA binding domains. In addition, recent studies suggest that occupancy of enhancer regions by nucleosomes may actually favor pioneer factor binding and thereby mediate cooperativity between factors that would not necessarily interact on naked DNA (Iwafuchi-Doi and Zaret, 2014; Iwafuchi-Doi et al., 2016).

But what if the nucleosome units are inaccessible, hidden in tight heterochromatin? For a number of pioneer TFs special chromatin binding properties have been reported. An example is the sequence-independent affinity of some bona-fide pioneer TFs to histone modifications, such as mono- or di-methylation of lysine 4 on histone 3 (H3K4^me1^ or H3K4^me2^), epigenetic modifications that occur on active enhancers (Cirillo et al., 2002; Sekiya et al., 2009; Magnani et al., 2011). The current view is that this initial, global recruitment to chromatin enables the pioneer TFs to scan the surrounding sequences for their recognition motives (Soufi et al., 2015). Other heterochromatin regions, like those where H3K9^me2^ or H3K9^me3^ marks are deposited, remain inaccessible even to pioneer factors (Soufi et al., 2012; Iwafuchi-Doi and Zaret, 2014). Collectively, these studies have begun to shed light onto the versatile mechanisms used by pioneer factors to engage their target sites in closed chromatin (Table 1). However, the sequence of events during initial heterochromatin opening, involving chromatin modifications, pioneer factors, and possibly additional components, are still subject of debate (Choukrallah and Matthias, 2014). In addition, mechanistic details are only known for a small number of pioneer factors at present. Given the enormous complexity of cell lineage decisions during embryonic development and the recent advancements to revert these decisions by cellular reprogramming strategies, many more TFs with pioneering activity may wait to be discovered.

**Table 1:** **Overview over pioneering mechanisms**.

**Pioneer factor**	**Cellular context of pioneering**	**Proposed pioneering mechanism**	**References**
Ascl1	- Direct lineage conversion from fibroblasts or other somatic cells to neurons - Neuronal differentiation from ESCs	Nucleosomal targets contain an extra “G” nucleotide at the 3′-end of the E - Box motif	Vierbuchen et al., 2010; Karow et al., 2012; Wapinski et al., 2013; Yamamizu et al., 2013; Raposo et al., 2015; Soufi et al., 2015
FoxD3	- Hepatocyte initial specification, - Binding to the *Alb1* enhancer in ESCs	Winged helix DBD	Clark et al., 1993; Ramakrishnan et al., 1993; Gualdi et al., 1996; Bossard and Zaret, 1998; Xu et al., 2007, 2009
FoxA1/A2	- Hepatocyte later specification and differentiation, replacement of FoxD3 at the *Alb1* enhancer in definite endoderm; - Breast cancer (regulation of the estrogen response)	- Winged helix DBD; - Favors “accessible nucleosomes” - Genome scanning - H3K4^me1^/H3K4^me2^ binding	Xu et al., 2007; Magnani et al., 2011; Magnani and Lupien, 2014; Iwafuchi-Doi et al., 2016
Klf4	- iPSC reprogramming, - Different types of cancer - Cell lineage specification in the embryo	- Targeting of a degenerate hexameric motif (instead of canonical nonameric motif) at nucleosome-enriched sites	Takahashi and Yamanaka, 2006; Soufi et al., 2012, 2015
Oct4	- iPSC reprogramming, - Several types of cancer - Involved in the development of different cell lineages during embryogenesis	- Binding of separate half sites of the bipartite POU-domain (POU_S_/POU_HD_)	Takahashi and Yamanaka, 2006; Soufi et al., 2012, 2015
PU.1	- Hematopoietic lineages	Unknown	Smale, 2010; Barozzi et al., 2014
Sox2	- iPSC reprogramming - Neuronal fate specification	Recognition of a degenerate motif facilitating recognition of histone bound DNA minor groove	Takahashi and Yamanaka, 2006; Karow et al., 2012, 2014; Soufi et al., 2015
Pbx1	- Skeletal muscle lineage specification; - Breast cancer (regulation of the estrogen response); - Adult neurogenesis and neuronal lineage specification	- H3K4^me1^/H3K4^me2^ binding	Berkes et al., 2004; Maves et al., 2007; Magnani et al., 2011; Thiaville et al., 2012; Grebbin et al., 2016

*Summarizes several established pioneer factors in comparison to PBX1. The list gives the physiological contexts in which priming was established and potential mechanisms that may mediate their priming function. For more detailed information on these proteins or for further discussion of general priming and pioneer factor mechanisms we would like to refer the reader to a series of excellent recent reviews (Iwafuchi-Doi and Zaret, 2014; Magnani and Lupien, 2014; Zaret and Mango, 2016)*.

## PBC proteins in development, adult stem cells, and cancer

Pre-B cell leukemia (PBC) TFs are evolutionarily conserved, atypical homeodomain proteins. Phylogenetically, they constitute one class of the *T*hree *A*mino acid *L*oop *E*xtension-homeodomain (TALE-HD) superclass, a separate branch of the homeodomain proteins characterized by the name-giving *T*hree *A*mino acid *L*oop *E*xtension (“TALE”) between the first and second alpha-helix of the homeodomain (Gehring et al., 1994; Bürglin and Affolter, 2016). In animals, TALE-HD proteins can be subdivided into five classes (PBC, MEIS, IRO, MKX, TGIF), of which the PBC-class, consisting of pre-B cell leukemic homeobox (PBX) 1–4 proteins in mammals, will be addressed in more detail here.

PBC proteins were originally identified as HOX-cofactors in *D. melanogaster*, because mutants of the fly PBC protein *extradenticle* displayed homeotic transformations similar to those seen in *Hox*-mutant animals, without altering the expression of the respective *Hox* genes themselves (Peifer and Wieschaus, 1990; Rauskolb et al., 1993, 1995). Subsequent studies revealed that this protein class contributes to the correct patterning of the anterior–posterior and proximal–distal body axes, confers regional identity in the embryo and is involved in the regulation of proliferation, apoptosis, and differentiation during embryogenesis (Berkes et al., 2004; Ferretti et al., 2011; Gordon et al., 2011; Koss et al., 2012; Yao et al., 2013). For instance, knockout of *Pbx1* in mouse embryos leads to embryonic lethality at E15/E16 with hypoplasia or aplasia of several organs, including impaired hematopoiesis, incomplete development of the thymus, spleen agenesis, pancreas hypoplasia, second branchial arch transformation, malformations of cervical vertebrae, ribs, and proximal limbs, and failure of septation of the cardiac outflow tract (DiMartino et al., 2001; Selleri et al., 2001; Manley et al., 2004; Brendolan et al., 2005; Stankunas et al., 2008). The unifying concept emerging from these studies is that PBX proteins act at or near the top of multiple cell fate hierarchies.

Corroborating their multifaceted roles during embryogenesis, dysregulation of PBC proteins is also a frequent phenomenon in cancer. In fact, the mammalian PBX1 protein was first identified in a chromosomal translocation [t(1;19) (q23;p13.3)] in pre-B cell acute lymphoblastic leukemia (ALL) that resulted in the expression of an oncogenic E2A-PBX1 fusion protein (Carroll et al., 1984; Williams et al., 1984; Kamps et al., 1990, 1991). Oncogenic roles of HOX/PBX dimers have also been reported in many other cancers and can be blocked by peptide-based inhibition (Morgan et al., 2007, 2010; Ando et al., 2014; Kelly et al., 2016). In addition, PBX1 plays an important role in estrogen receptor alpha (ERα)-positive breast carcinogenesis (Magnani et al., 2011). In contrast to their rather well-studied roles in embryonic development and cancerous malignancies, the contribution of PBC proteins to adult stem cell niches is still largely unexplored. In the hematopoietic system, PBX1 regulates long term hematopoietic stem cell quiescence, limits myeloid maturation and preserves a lymphoid potential in multipotent progenitor (MPP) and common myeloid progenitor (CMP) pools, thereby regulating and maintaining progenitor reservoirs (Ficara et al., 2013).

The subventricular zone (SVZ) in rodents is an adult stem cell niche that provides new neurons and glia to the brain. In brief, adult stem cells residing in the SVZ produce young neurons, termed neuroblasts, via an intermediate population of transient amplifying progenitor cells. Neuroblasts leave the SVZ and migrate into the olfactory bulb where they terminally differentiate to distinct types of interneurons that are continuously replaced in the existing circuitry as part of a life-long remodeling of the olfactory system. A number of TFs, including DLX2 and PAX6, bias progenitor cells toward a general neuronal fate and promote their subsequent maturation to defined types of interneurons (Hack et al., 2005; Brill et al., 2008). In cooperation with PAX6 and DLX2, the TALE-HD protein MEIS2 regulates neuronal cell fate acquisition, as well as the terminal differentiation of neuroblasts into dopaminergic periglomerular neurons (Agoston et al., 2014). Recently, we characterized the contribution of PBX1 to adult SVZ neurogenesis (Grebbin et al., 2016). We observed high PBX1 expression in rapidly proliferating SVZ progenitors and neuroblasts, as well as in subsets of their progenies in the olfactory bulb, including dopaminergic neurons. Targeted deletion of *Pbx1* in transient amplifying progenitor cells (in a *Pbx2*-deficient background to prevent functional compensation by this structurally related gene) significantly reduced the production of neurons and increased the generation of oligodendrocytes *in vitro* and *in vivo*, establishing Pbx1 as an early lineage regulator of SVZ neurogenesis. Loss of *Pbx1* expression in neuronally committed neuroblasts, by contrast, severely compromised cell survival. By chromatin immunoprecipitation from endogenous tissues or isolated cells, we identified the neuron-specific gene *doublecortin (Dcx)* and the dopaminergic neuron marker gene *tyrosine hydroxylase (Th)*, as direct PBX1 target genes.

Notably, PBX1 binds to its target sites in promoter/enhancer regions of these genes already in undifferentiated progenitor cells and hence at times that significantly precede the transcriptional activation of both genes. The *Dcx* gene encodes a microtubule-associated protein, which is expressed by all migrating neuroblasts and therefore a frequently used marker for young neurons (Francis et al., 1999; Gleeson et al., 1999). Primary cultures of proliferating adult SVZ neural stem- and progenitor cells (neurospheres) are DCX negative, but the protein becomes quickly upregulated once differentiation is induced. Although undifferentiated neurospheres do not yet express DCX and the *Dcx* locus exhibits very low levels of the activating epigenetic histone modification H3K4^me3^, a significant enrichment of PBX1 at the *Dcx* promoter was observed, indicating that PBX chromatin binding preceded *Dcx* gene activation. In contrast to the relatively fast upregulation of pan-neuronal markers like *Dcx*, terminal differentiation and integration of adult generated neurons into the existing circuitry of the olfactory bulb represent the last steps of neuronal turnover during adult neurogenesis. Full maturation of dopaminergic neurons is a particularly slow process and ~2 months pass before an adult SVZ-generated neuron reaches the olfactory bulb and achieves a level of cellular maturation at which *Th* expression is initiated (Brill et al., 2008). Unexpectedly, PBX1 occupies the *Th* promoter/proximal enhancer already in progenitor cells and newborn neuroblasts in the SVZ, suggesting that priming by PBX1 can precede transcriptional activation of the *Th* gene by several months.

## PBX pioneering?

Our observation that PBX1 may bind its targets in silent gene loci does not stand alone. In fact, the first evidence for a pioneering role of PBX1 was provided by Berkes et al. (2004). The authors observed that during skeletal muscle differentiation PBX1 is constitutively bound to the promoter of the *Myogenin* gene. Differentiation and activation of *Myogenin* expression are subsequently initiated by the pro-myogenic transcription factor MYOD through interaction with pre-bound PBX1. At the onset of myogenic differentiation, PBX1 thus seems to serve as a platform for MYOD binding in inactive chromatin, thereby preparing genes of the skeletal muscle lineage for activation (Berkes et al., 2004; Maves et al., 2007). This finding was supported in 2011 by a report describing PBX1 as a pioneer factor in ERα-signaling in breast cancer (Magnani et al., 2011). Using the cell line MCF7 as model to investigate the activation of oncogenic ERα target genes during breast cancer progression, the study identified PBX1 as “partner” pioneer factor to FOXA1. PBX1 is pre-bound to shared PBX1-ERα binding sites proximal to genes involved in cancer cell proliferation prior to estrogen application and its binding to these sites remains following estrogen treatment. Although FOXA1 and PBX1 pioneering functions are independent from each other, the presence of both factors has a synergistic effect on chromatin openness on shared binding sites (Magnani et al., 2011). Together, PBX1 pre-loading to chromatin before estrogen treatment and its ability to induce chromatin opening argue for a pioneering function in this context.

In contrast to FOXA1, little is known about the mechanisms that may allow PBX1 to access silent chromatin. However, the detailed structural analysis of the iPSC reprogramming factors Oct4, Sox2, Klf4, and c-Myc may provide a hint. Specifically, the length of the basic helix-loop-helix domain of bHLH proteins appears to inversely correlate with pioneer activity, as short bHLH basic regions facilitate nucleosomal DNA binding, whereas proteins with longer bHLH basic regions depend on the cooperation with other pioneer factors (Nair and Burley, 2003; Sauvé et al., 2004; Soufi et al., 2015). The ability of a bHLH protein to access closed chromatin thereby appears to be either defined by the way helix 1 contacts DNA or by cooperation with additional factors, leading to recognition of target sequences which may contain partial, degenerate or altered motifs (Soufi et al., 2015). In this context it is intriguing to consider that the TALE-homeodomain, owing to the insertion of three amino acids between helix 1 and helix 2, is structurally distinct from other homeodomains (Gehring et al., 1994; Bürglin and Affolter, 2016). This raises the possibility that the TALE-HD might be especially suited to recognize its consensus motif on nucleosomal DNA, but to date mechanistic details remain unknown.

An alternative scenario was described by Magnani et al. who reported that PBX1, as had been previously shown for FOXA1, preferably associates with nucleosomes carrying H3K4^me1^ and H3K4^me2^ modifications (Magnani et al., 2011; Sérandour et al., 2011; Jozwik et al., 2016). Mono- and di-methylation of H3K4 mark distal enhancers that are linked to active or poised genes and can be found as biochemical intermediates preceding the triple-methylation of H3K4 at transcriptionally active promoters. As H3K4^me1^ deposition is catalyzed by the Set/MLL family proteins MLL3 and MLL4, this would paradoxically suggest that FOXA1 and PBX1 binding to DNA requires prior histone modification by MLL3/4, a notion that is difficult to reconcile with the proven or proposed pioneering activity of FOXA1 and PBX1, respectively (Hu et al., 2013). A possible explanation comes from the observation that the relationship between pioneer factor binding and H3K4 epigenetic modification seems to be bidirectional: The pioneer factors FOXA1 and PU.1 not only preferentially bind to chromatin carrying H3K4^me1^/H3K4^me2^ modifications, but their association in turn promotes H3K4^me1/2^ deposition (Heinz et al., 2010; Sérandour et al., 2011). The essential question of which is present on the chromatin first, the nucleosomal modification or the pioneer factor, cannot be conclusively answered. At present, examples for both cases exist and quite possibly, pioneer factor binding and epigenetic histone modifications stabilize each other. Moreover, unknown additional players might be involved, such as long or short non-coding RNAs. In any respect, further efforts are needed to decipher the whole spectrum of mechanisms used by pioneer factors to access closed chromatin.

In conclusion, multiple lines of evidence, obtained from *in vivo* and *in vitro* studies and made in the context of embryonic development, adult stem cell differentiation and cancer, suggest that PBX1 may act as pioneer factor. The three hallmarks of pioneer factor function—target site binding in closed chromatin, the ability to increase DNA access for other proteins and active involvement in cell fate specification or cellular (re-)programming—have all been demonstrated for PBX1. Yet, each of these features has been investigated in a different physiological setting and with different tools. A unifying model is therefore still missing. In addition, little is known about whether the other three members of the mammalian PBC family, PBX2-4, also possess the ability to recognize their target sites in silent chromatin or even pioneer factor activity. A coherent assessment of PBX pioneering function by standardized approaches is therefore an important next step in research on this protein family.

## Author contributions

DS and BG jointly wrote the manuscript and approved it for publication.

## Funding

Research related to this Review was funded by the Deutsche Forschungsgemeinschaft, grant SCHU1218/3-1 to DS and a Ludwig Edinger Fellowship to BG.

### Conflict of interest statement

The authors declare that the research was conducted in the absence of any commercial or financial relationships that could be construed as a potential conflict of interest. The reviewer CC and handling Editor declared their shared affiliation, and the handling Editor states that the process nevertheless met the standards of a fair and objective review.
